# Retraction: Pyrroloquinoline Quinine Inhibits RANKL-Mediated Expression of NFATc1 in Part via Suppression of c-Fos in Mouse Bone Marrow Cells and Inhibits Wear Particle-Induced Osteolysis in Mice

**DOI:** 10.1371/journal.pone.0360868

**Published:** 2026-10-19

**Authors:** The PLOS One Editors

Following the publication of this article, concerns were raised with the results presented in Figs 1, 2, and 5. Specifically,

- There appear to be multiple repetitive features within and/or between all Fig 1A panels.
- The Fig 2D NFATc1 panel of [1] appears similar to the Fig 3B NFATc1 panel of [2] despite being used to represent different experimental conditions.
- The following panels appear to partially overlap despite being used to represent different experimental conditions:
  - Fig 5a of [1] and Fig 4a of [3] when flipped.
  - Fig 5b of [1] and Fig 4b of [3].
  - Fig 5c of [1] and Fig 4d of [3] when flipped.
  - Fig 5e of [1] and Fig 4e of [3].
  - Fig 5f of [1] and Fig 4f of [3].
  - Figs 5i and 5k of [1].

In light of these concerns, which call into question the integrity and reliability of the published results, the *PLOS One* Editors retract this article.

All authors either did not respond directly or could not be reached.
